# The Anæsthetic Effects of Cold Water

**Published:** 1877-03

**Authors:** 


### THE ANÆSTHETIC EFFECTS OF COLD WATER.

Several writers have claimed to have witnessed remarkable anæsthetic effects from hypodermic injections of pure cold water. Among these, the inventor of the aspirator, Dr. Dieulafoy, has, for several years past, been in the habit, in acute articular rheumatism, of injecting some ten drops of cold water around different parts of the affected joint, as a means of relieving the pain. The results are most remarkable. The pains abate, and the patient is enabled to move the joint; and in some cases the rheumatism is even cured by this simple means. The same means may be employed als in muscular rheumatism, ischias, etc. A repetition of these experiments does not always bring the same satisfactory results. —*Med. & Surg. Reporter.*

